# Music practice is associated with development of working memory during childhood and adolescence

**DOI:** 10.3389/fnhum.2013.00926

**Published:** 2014-01-07

**Authors:** Sissela Bergman Nutley, Fahimeh Darki, Torkel Klingberg

**Affiliations:** Neuroscience Department, Developmental Cognitive Neuroscience, Karolinska InstitutetStockholm, Sweden

**Keywords:** musical practice, working memory, reasoning, cognitive development, gray matter volume

## Abstract

Practicing a musical instrument is associated with cognitive benefits and structural brain changes in correlational and interventional trials; however, the effect of musical training on cognition during childhood is still unclear. In this longitudinal study of child development we analyzed the association between musical practice and performance on reasoning, processing speed and working memory (WM) during development. Subjects (*n* = 352) between the ages of 6 and 25 years participated in neuropsychological assessments and neuroimaging investigations (*n* = 64) on two or three occasions, 2 years apart. Mixed model regression showed that musical practice had an overall positive association with WM capacity (visuo-spatial WM, *F* = 4.59, *p* = 0.033, verbal WM, *F* = 9.69, *p* = 0.002), processing speed, (*F* = 4.91, *p* = 0.027) and reasoning (Raven’s progressive matrices, *F* = 28.34, *p* < 0.001) across all three time points, after correcting for the effect of parental education and other after school activities. Music players also had larger gray matter volume in the temporo-occipital and insular cortex (*p* = 0.008), areas previously reported to be related to musical notation reading. The change in WM between the time points was proportional to the weekly hours spent on music practice for both WM tests (VSWM, β = 0.351, *p* = 0.003, verbal WM, β = 0.261, *p* = 0.006) but this was not significant for reasoning ability (β = 0.021, *p* = 0.090). These effects remained when controlling for parental education and other after school activities. In conclusion, these results indicate that music practice positively affects WM development and support the importance of practice for the development of WM during childhood and adolescence.

## INTRODUCTION

Previous research on practice on specific skills demonstrates domain specific expertise, whether it be within the field of chess, memorizing numbers, or dance, with little or no transfer evident of this superiority to other tasks (Ericsson and Lehmann, 1996). This is explained by the use of material specific strategies, schemas, and automatization of the procedures being performed (Ericsson et al., 2006). However, the development of such strategies requires abilities considered to be executive functions such as working memory (WM), updating, monitoring of performance, etc. In the emerging field of cognitive training, targeted interventions aim to train these types of cognitive abilities, with WM being the most studied to date. The most effective of these paradigms often shows large effects on the trained ability and moderate effects on closely related abilities after as little as 5 weeks of computerized training (for a review, see Klingberg, 2010). This is of importance for theoretical and clinical reasons where a particular ability is deficient. A different way of achieving cognitive enhancement may be to target the entire bodily system through physical training where such effects have been observed (Hillman et al., 2008; Hotting and Roder, 2013). Yet another way to achieve this may be to regularly engage in a complex activity that requires one to use higher order thinking, such as playing a musical instrument.

Formal music practice involves several cognitively challenging elements, e.g., long periods of controlled attention, keeping musical passages in WM or encoding them into long-term memory, decoding music scores, and translating the product into corresponding motor commands. This type of activity taxes complex cognitive functions as seen in brain imaging research (Schon et al., 2002; Stewart et al., 2003). Other investigations suggest that music practice is associated with cognitive benefits (for a review, see Schellenberg and Weiss, 2013).

Associations between formal music training and cognitive ability have mostly been reported in retrospective studies of musicians and non-musicians (Schellenberg, 2006; Forgeard et al., 2008; Ruthsatz et al., 2008). Individuals practicing music demonstrate higher performance in tasks requiring visuo-spatial reasoning (Hurwitz et al., 1975; Rauscher et al., 1997; Bilhartz et al., 1999; Costa-Giomi, 1999; Schellenberg, 2004, 2006; Forgeard et al., 2008; Ruthsatz et al., 2008), processing speed (Schellenberg, 2006; Bugos et al., 2007) as well as WM (Schellenberg, 2006; Bugos et al., 2007; Pallesen et al., 2010). These effects point to a quite general cognitive advantage for music players compared with non-music players. There are also reports of associations between the number of months of music practice and academic performance in math, reading and spelling after controlling for general intelligence and parental education (Schellenberg, 2006), although these findings are not always consistent (Forgeard et al., 2008). A recent study also reported a positive association between music practice and grades in practically all school subjects in three separate grades (Cabanac et al., 2013). A reoccurring question in retrospective correlation studies is whether the differences reported in musically trained individuals predate their music training or appear as a consequence of it. There are however, a few prospective randomized controlled studies suggesting a causal relation between music and cognitive ability demonstrated with measures of processing speed and WM (Bugos et al., 2007) as well as on full scale IQ (Schellenberg, 2004).

Extensive music training is known to affect the anatomy of the brain, with greater gray matter volumes observed in motor-related areas (Elbert et al., 1995; Pascual-Leone, 2001; Hyde et al., 2009), auditory discrimination areas (Gaser and Schlaug, 2003; Hyde et al., 2009) as well as greater white matter volumes in motor tracts (Bengtsson et al., 2005) in professional musicians. Whilst few studies have been carried out to understand the neurophysiological effects of music training on cognitive abilities there is some evidence to suggest an absence of pre-existing difference in music-related brain functions prior to learning an instrument (Norton et al., 2005), with differences emerging approximately 1 year after music training has commenced (Schlaug et al., 2005; Hyde et al., 2009).

Over the past decade, it has been clearly demonstrated that certain cognitive abilities are susceptible to targeted training. For instance, WM capacity has been shown to improve with as little as 5 weeks of cognitive training (Klingberg et al., 2002, 2005; Westerberg et al., 2007; Holmes et al., 2009; Thorell et al., 2009; Bergman Nutley et al., 2011; Brehmer et al., 2012; Green et al., 2012). The neural effects of this type of training have been studied with functional magnetic resonance imaging (fMRI), revealing increased prefrontal and parietal activity (Hempel et al., 2004; Olesen et al., 2004; Jolles et al., 2010). Other studies have reported gains in fluid intelligence after training WM (Klingberg et al., 2002, 2005; Jaeggi et al., 2008) or non-verbal reasoning (Bergman Nutley et al., 2011; Mackey et al., 2011), although the transfer effects from WM to reasoning tests are smaller and inconsistent across studies compared to the effect on WM (Holmes et al., 2009; Thorell et al., 2009). Studies also show that processing speed is susceptible to computerized training improvements (Mackey et al., 2011; Wolinsky et al., 2013). There is also emerging evidence, although with mixed results thus far, that WM training may affect academic performance with improvements in mathematical problem solving (Holmes et al., 2009) and reading comprehension reported (Dahlin, 2010; Egeland et al., 2013). If causality can be established between music training and its association with cognitive and academic benefits, then music training should perhaps be considered a type of cognitive training.

To date, the specific aspects of musical training that affect cognitive improvements have not been identified. One candidate factor is the practice of sight reading. The complexity of sight reading requires rapid (and for some instruments dual) information processing, visuo-spatial decoding, and constant updating of notes to come while playing current notes. In other words, sight reading requires visuo-spatial WM abilities. Sight reading shows correlations with both IQ (*r* = 0.6; Salis, 1978) and with WM (*r* = 0.3–0.4; Salis, 1978; Meinz and Hambrick, 2010). Brain activation studies during sight reading have shown occipito-temporal and parietal activations (Nakada et al., 1998; Schon et al., 2002; Stewart et al., 2003; Bengtsson and Ullen, 2006), areas typically involved in visuo-spatial abilities (dorsal-stream) and pattern recognition.

While data suggest that there is a link between music practice and cognitive ability, it has not been ascertained how music training affects cognitive abilities during development. One factor expected to be of importance for the effect is the time spent training (Schellenberg, 2006; Forgeard et al., 2008), although some studies have failed to find a relation between the number of music lessons and cognitive ability (Ruthsatz et al., 2008; Meinz and Hambrick, 2010).

Most studies have thus far either had small sample sizes (Rauscher et al., 1997; Gromko and Poorman, 1998; Bilhartz et al., 1999; Schlaug et al., 2005; Bugos et al., 2007) or have simply correlated music and cognitive ability in natural samples at one time point providing no information about causality (Schellenberg, 2006; Forgeard et al., 2008; Ruthsatz et al., 2008). To test if music training is associated with differential trajectories of the development of different cognitive functions, longitudinal designs on developing populations could be useful. This study aimed to investigate the effects of music training on WM, processing speed, and reasoning ability in a longitudinal developmental sample.

By using a longitudinal approach we can study the change in cognitive ability related to musical practice, controlling for initial cognitive performance, age, and other possible confounders. More specifically we hypothesize that music training will be associated with: (1) over all positive cognitive effects as seen in performance on neuropsychological tests of WM, processing speed and reasoning ability (primary outcomes) as well as on tests of mathematical and reading ability (secondary outcomes) across time points, (2) a dose-related development of cognitive and academic ability (as seen with the same outcomes as in hypothesis 1) between time points, i.e., a positive linear relation between the amount of practice and the magnitude of the cognitive and academic benefits, (3) structural differences in the brain between music players and non-music players across the three time points.

## MATERIALS AND METHODS

### PARTICIPANTS

This study used a longitudinal design with three completed measurement points, T1, T2, and T3 collected in 2007, 2009, and 2011, respectively. Participants between the ages of 6 and 25 were randomly chosen from the population registry in the town of Nynäshamn, Sweden. At each time point, information regarding the study was sent out to the parents together with a consent form to be returned to the researchers upon agreement. Parents were then contacted by telephone and given an opportunity to ask questions. Participants aged 18 years and older were contacted directly. At T1, 339 subjects participated in the study, out of which 273 also participated at T2 and 65 at T3. Exclusion criteria were: a first language other than Swedish, any diagnosis of psychiatric or neurological disorder (with exception to ADHD or dyslexia), any vision or hearing impairment considered to affect the test-performance. The study was approved by the local ethics committee in Stockholm. A subset of the sample where also asked to participate in neuroimaging data collection (see **Table 1** for sample sizes).

**Table 1:** Sample sizes and descriptive data for the variables included for each time point, with standard deviations in parenthesis.

Time	1		2		3	
Group	No music	Music	No music	Music	No music	Music
Total (*N*)	280	59	225	48	48	17
Females (*N*)	135	33	110	30	22	10
Neuroimaging (*N*)	64	0	56	9	48	13
Music (h/week)	0	3.66 (4.98)	0	4.29 (4.35)	0	5.06 (4.98)
Physical activity (h/week)	1.00 (2.17)	1.42 (2.25)	1.41 (2.46)	1.29 (2.13)	1.71 (2.76)	1.18 (2.56)
TV/online/gaming (h/week)	20.14 (15.61)	20.29 (13.61)	26.34 (17.09)	24.31 (19.73)	33.48 (21.78)	23.47 (17.58)
Mother edu (years)	12.79 (2.39)	12.78 (2.26)	12.83 (2.28)	13.52 (2.52)	12.59 (2.47)	14.00 (2.82)
Father edu (years)	12.03 (1.92)	12.11 (2.28)	12.09 (2.15)	12.71 (2.75)	12.34 (2.52)	12.89 (3.65)
Age (years)	12.01 (4.49)	13.39 (4.38)	13.68 (4.05)	13.59 (3.92)	16.8 (4.69)	15.64 (5.76)
Dot matrix (raw score)	25.38 (7.44)	28.22 (5.95)	27.93 (7.31)	29.60 (6.55)	30.44 (6.46)	33.53 (6.88)
Digit recall (raw score)	13.98 (5.61)	16.50 (6.61)	15.99 (5.64)	16.81 (5.58)	17.94 (5.14)	20.53 (7.81)
Letters digit (raw score)	22.37 (12.16)	26.73 (11.87)	27.81 (10.77)	28.65 (11.50)	32.64 (9.45)	31.35 (10.87)
Raven (standardized)	-0.13 (0.87)	0.44 (1.02)	0.18 (0.80)	0.42 (0.82)	0.50 (0.71)	0.70 (1.06)
Math (standardized)*	-0.31 (0.91)	0.22 (0.92)	-0.19 (0.99)	-0.02 (0.97)	0.41 (0.71)	0.49 (0.81)
Reading (standardized)*	-0.15 (0.97)	0.23 (0.86)	-0.05 (1.01)	0.16 (0.87)	0.49 (0.57)	0.46 (0.55)

^*^The academic tests were assessed through ages 8–25 and thus the *N*s for the table do not encompass these two variables but had a total of 273 at T1.

### COGNITIVE TESTS

Neuropsychological assessment and questionnaires were administered at T1, T2, and T3. The tests were administered individually in different schools in a separate, quiet room. The questionnaire was sent to the participant’s homes, covering information regarding after school activities and socio-economic background. Raw data from the assessments are found in **Table 1**.

In order to assess visuo-spatial WM, the Dot matrix from the Automated Working Memory Assessment (AWMA) battery was used (Alloway, 2007). This is a simple WM task and involves remembering the location and order of dots displayed sequentially in a grid on a computer screen. The dots were displayed in red in a four-by-four grid on a white background. Each dot was presented for 1000 ms, with an intra-stimulus interval of 500 ms. Each level consisted of six trials, with four correct trials as the minimum required for moving to the next level, where the number of dots to be remembered increased by one item. The test terminated after three errors were committed on one level. The Dot matrix test was performed on a HP Compaq nc6320 laptop with a 15-in screen.

Backward digit recall was used to assess verbal WM (Alloway, 2007). This test requires the participant to repeat a list of numbers read out loud in reverse order and started with two digits. Difficulty level increased until the participant failed to pass a level. Each level consisted of six trials. Four correct trials were required to move on to the next level.

Speed of processing was measured using a letter–digit substitution task. In this task, the participants were shown a row with nine consonants, each paired with a number. Underneath, nine additional rows of 15 letters were presented with the numbers absent. The task was to pair as many numbers with their corresponding letter as possible during a 1 min period.

Raven’s Advanced Progressive Matrices (Raven, 2003) was used to assess non-verbal reasoning. The test consists of matrices in black and white distributed over sets A–D for the 6-year-olds and A–E for the rest, with 12 items in each set. The task was to identify the completing piece to a matrix, having a choice of 6 in sets A–B and a choice of 8 in sets C–E. Because the items performed differed between individuals in the sample the outcome underwent item response theory modeling where the performance of each participant was converted into a standardized score which was used as the outcome measure.

### ACADEMIC TESTS (AGES 8–25)

The arithmetical assessment was based on the Trends in Mathematics and Science Study (Martin et al., 2004) and Basic Number Screening Test (Gillham and Hesse, 2001), having been designed for four school-grade-dependent versions (grades 2, 4, 6, and 8, suitable for 14–27-year-olds). Grades 2 and 4 problems included magnitude judgments, questions about the number sequence, as well as elementary arithmetic (addition, subtraction, division, multiplication, and fractions). Grades 6 and 8 problems included elementary arithmetic and elementary algebra (simple equations with variables).

Narrative and expository texts from the Progress in International Reading Literacy Trend Study (PIRLS 2001 T) and The International Association for the Evaluation of Educational Achievement Reading Literacy Study 1991 (Gustafsson and Rosén, 2004) were used to measure reading comprehension. Seventy-seven items were used to form four age-adapted reading comprehension tests for 8–25 years olds.

### LIFE STYLE QUESTIONNAIRE

The questionnaire included questions on musical practice (yes/no), the instrument played, and the number of hours per week of practice. It also included questions on the highest level of education for each parent (total years of education used as a continuous variable), time spent online, gaming and watching TV (summed together in analyses) and time spent doing physical activity outside of physical education.

For the first hypothesis the music practice was coded as a binary variable (0/1) in order to investigate the relation between music practice and cognitive and academic performance overall (across the time points). The second hypothesis was investigated using the number of weekly hours reported spent on music practice for the time point previous to the predicted one as an explanatory variable (for individuals who had reported playing for two of the time points) for cognitive and academic development.

### NEUROIMAGING

Three-dimensional structural T1-weighted imaging (magnetization-prepared rapid gradient echo sequence, repetition time 2300 msec, echo time 2.92 msec) with a 256 mm × 256 mm field of view, 176 sagittal slices, and 1 mm^3^ voxel size was carried out with a 1.5T Siemens Avanto scanner on 64 participants and repeated after 2 and 4 years. GRAPPA parallel imaging technique with an acceleration factor of two was also employed to speed up the acquisition. Gray matter segmentation was performed on the structural data with a voxel-based morphometry tool available via SPM5 (www.fil.ion.ucl.ac.uk/spm/software/spm5) and followed by an alignment technique performed with the Diffeomorphic Anatomical Registration with Exponentiated Lie algebra (DARTEL) toolbox in SPM. This method iteratively aligned the gray matter images from all three time points to their common average template. The modulated images were then spatially smoothed with a Gaussian kernel size of 8 mm and registered to Montreal Neurological Institute space. Because the DARTEL morphing was applied to tissue segmented images, output images were the tissue probability maps in which each voxel shows the probability of being locally expanded or contracted.

## RESULTS

Descriptive information regarding the demographics of the sample is included in **Table 1**. There was a significant difference between groups (music vs. non-music) in age (*t* = 1.98, *p* = 0.048) at time point 1, and trends for maternal and paternal education levels [*t* = 1.91, *p* = 0.061 (T3) and *t* = 1.77, *p* = 0.077 (T2)], and time spent online/gaming/TV viewing at T3 (*t* = 1.89, *p* = 0.067; as tested with *t*-tests of independent samples at each time point). There was neither a difference between the music and non-music players in weekly hours spent on physical exercise (*t* < 1.36, *p* > 0.17), nor on any of the other covariates at the remaining time points (all *t*s < 0.71, *p* > 0.48).

In order to investigate the first hypothesis: whether music practice was associated with a different performance level on the four cognitive tests across all three time points, we ran mixed linear regression models with time as the repeated variable (unstructured repeated covariance type) performance on the four tests, respectively, as the dependent variables with sex, time, and music practice (yes/no) as the factors and age (inverse), father’s and mother’s education levels (total years), hours spent on gaming/internet/TV viewing and on physical activity as covariates.

**FIGURE 1:**
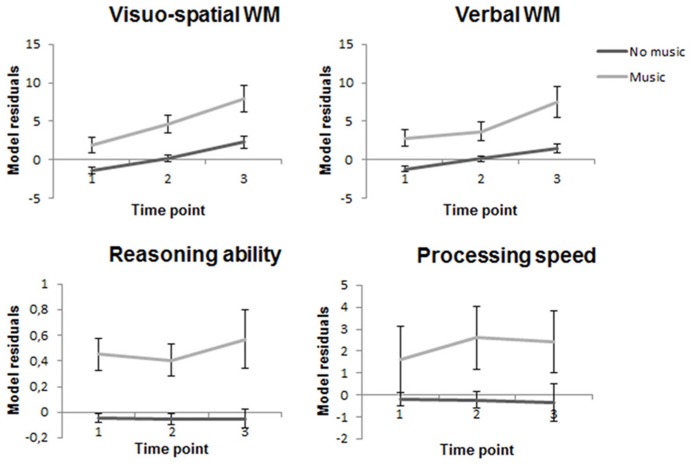
**Cognitive performance across the three time points with the residuals from mixed models (with age, sex, time, parental education level, time spent on physical activity, gaming/internet/TV viewing as independent variables) for the primary outcomes plotted according to music training category.** Error bars represent standard error of the mean (SEM).

There was a significant main effect of music on visuo-spatial WM [*F*(1,333) = 4.59, *p* = 0.033], verbal WM performance [*F*(1,333) = 9.69, *p* = 0.002] processing speed [*F*(1,333) = 4.91, *p* = 0.027], and reasoning ability [*F*(1,332) = 28.34, *p* < 0.001]. There was also a significant positive association between music practice and math performance [*F*(1,317) = 12.91, *p* < 0.001] but not with reading comprehension [*F*(1,317) = 1.42, *p* = 0.23]. The residuals from corresponding mixed models excluding the music factor are plotted for the primary outcomes according to music category (thus removing the effect of age, sex, time, parental education, and hobbies) in **Figure 1**.

Next, we evaluated hypothesis number two: the expected dose-related development of cognitive ability between time points. This was done by running a mixed linear regression model (unstructured repeated covariance type) with a variable for later time points (e.g., visuo-spatial WM Late, which could be either data from T2 or T3 or both if the subject participated at all three time points) as the dependent variable and a variable for earlier time points (e.g., visuo-spatial WM Early, which could be T1 or T2 or both) as independent variables so that data from individuals participating in two or three time points could be included as repeated measures. The models also included music hours/week, age (inverse), sex, father’s education level, mother’s education level, TV/online/gaming hours/week, and exercise hours/week as independent variables.

The results showed a significant effect of music practice hours/week on the outcome measures visuo-spatial WM, verbal WM with a trend for reasoning ability (see **Table 2** for regression coefficients). There was no significant effect of music practice hours/week on the development of processing speed, math, or reading performance (all βs for the music variable <0.151, *p* > 0.17).

**Table 2:** Regression coefficients for the models investigating the amount of change in cognitive performance on the three outcomes that could be explained by music hours/week and other possible factors.

	Dependent variables and model summaries		
	Visuo-spatial WM (late)		Verbal WM (late)		Reasoning (late)	
Independent variables	β	*P*	β	*P*	β	*P*
Constant	–	0.002	–	0.032	–	0.171
Sex	-0.568	0.242	0.300	0.422	-0.025	0.612
Age (Inverse)	-150.70	0.241	57.692	0.517	-20.47	0.145
Mother Edu (years)	0.048	0.680	-0.107	0.242	0.018	0.139
Father Edu (years)	0.185	0.138	0.043	0.653	-0.010	0.423
Outcome (Early)	0.715	<0.001	0.773	<0.001	0.668	<0.001
Online/gaming/TV(h/w)	-0.002	0.907	0.043	0.004	0.001	0.436
Exercise (h/w)	-0.229	0.078	0.157	0.110	-0.005	0.712
Music (h/w)	0.351	0.003	0.261	0.006	0.021	0.090

We investigated the presence of developmental windows where music practice would lead to larger cognitive effects by adding interaction terms of music practice by age to the models listed in **Table 2** but found no evidence of such an effect (with *p*s *>* 0.16).

**FIGURE 2:**
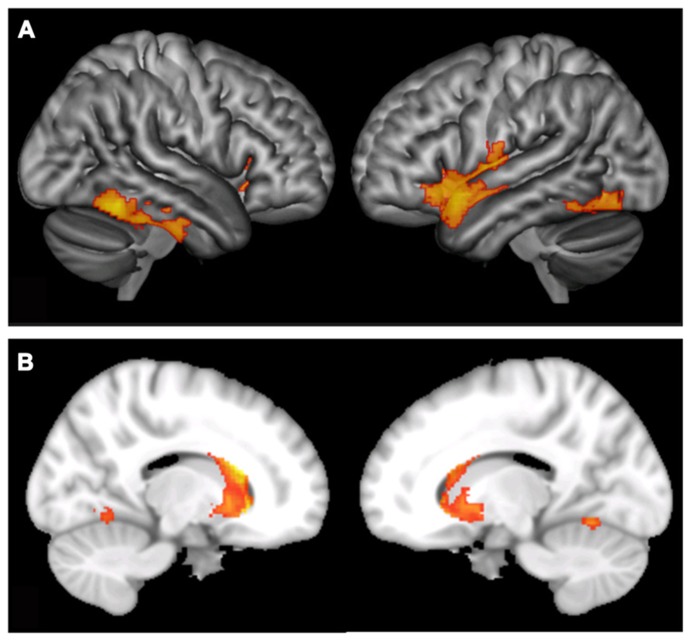
**The main effect of playing a musical instrument on gray matter volume; (A) effects in temporal cortex, occipito-temporal area, and the insula; **(B)** effects in caudate nucleus shown in sagittal section from both left and right hemispheres**.

The third hypothesis was that there would be a difference detected between music and non-music players in the brain. In order to find gray matter structural differences between music and non-music groups, we entered the music group as the main factor in a flexible factorial design analysis using SPM. All three time points of data were included in the analysis considering the subjects and time as factors, to take into account the repeated measures. The analysis was corrected for the effects of age, sex, handedness, and total gray matter volume. We also added an interaction term for age by group in order to detect age sensitive periods for music practice in the brain. The significant level was set at the cluster level with the threshold of *p* < 0.05 (corrected for the multiple comparisons of all voxels within gray matter volume). We found two clusters significantly showing differences in gray matter volume. One in the temporal lobe (*p* = 0.008, see **Figure 2A**) situated mainly in the inferior temporal and temporal-occipital fusiform gyri, based on the MNI structural and Harvard-Oxford Cortical Structural atlases (see **Table 3** for coordinates and cluster sizes), and the other cluster was in the insula, caudate, and putamen (*p* = 0.002 see **Figure 2B**). The interaction of age by group was not significant.

**Table 3:** Significant association between playing a musical instrument and gray matter volume.

Clusters overlap with*	*P* corrected cluster level	Number of voxels	*Z* score	*x*, *y*, *z* (MNI) for the peak voxel
Temporo-occipital fusiform gyrus, Inferior temporal gyrus	0.008	4408	3.96	34, -51, -9
Insula, caudate, and putamen	0.002	5337	3.64	20, 18, 17

^*^The labeling is based on both MNI structural and Harvard-Oxford Cortical Structural atlases provided by FMRIB Software Library (FSL) (http://fsl.fmrib.ox.ac.uk/fsl/fslwiki/Atlases).

## DISCUSSION

In this study, the effect of musical practice on cognitive ability was investigated in a developmental longitudinal study. The results showed that practicing a musical instrument was associated with higher performance on tests of reasoning, processing speed, and WM, as well as mathematics but not reading comprehension. Importantly, the development between time points was related to the time spent on music practice per week for both WM tests. The effect was also seen after correcting for baseline performance, parental education, and other after school activities, including physical activity, TV-watching, and gaming. These results confirm previously reported associations between musical practice and cognitive ability (Hurwitz et al., 1975; Schellenberg, 2006; Forgeard et al., 2008; Ruthsatz et al., 2008). The dose-response relation also supports the previously reported causal relation between the two (Schellenberg, 2004; Bugos et al., 2007).

We did not find a dose-response relation between music practice and the development of processing speed and only found a trend for the development of reasoning ability. The positive effects on the secondary outcome of mathematics did not point to a causal effect of music on academic development, and was only seen as an association with music practice across the time points. This is in line with previous findings of associations from correlational studies between music practice and grades in mathematics (Schellenberg, 2006; Cabanac et al., 2013) and mathematical performance (Schellenberg, 2006). As most of the music players in our sample were already practicing an instrument at the beginning of the study, it is possible that potential *pre-study* music-related changes in the development of WM underlie the learning advantages seen for mathematics, given its strong relation to visuo-spatial WM in particular (Bull et al., 2008). We did not see an association between music practice and reading comprehension, a finding previously reported in a correlational study (Schellenberg, 2006). The reason for this is unclear and should be further studied.

Differences were also seen in the gray matter volume of the brain between music players and non-players in the temporo-occipital and insular cortex across the time points. One could argue that since the music group outperformed the non-music group on cognitive measures at the beginning of the study that these differences should be controlled for in the brain imaging analysis. However, it is not certain and perhaps, given the literature (e.g., Schlaug et al., 2005), even unlikely that the cognitive differences are independent of music, thus controlling for them would also remove cognitive differences associated with music in the brain. The differences observed in the brain are consistent with previous findings showing higher gray matter volume for musicians in the fusiform gyrus (James et al., 2013), an area typically involved in visual pattern recognition. Specifically, this area has been identified through fMRI investigations as important during musical notation decoding (Schon et al., 2002; Stewart et al., 2003). These regions do not include the fronto-parietal networks involved in top-down attention and WM (Olesen et al., 2004). However, the effect was seen in the caudate nucleus which also has been implicated in WM training (Olesen et al., 2004; Dahlin et al., 2008; Backman et al., 2011). This demonstrates certain similarities between the neural correlates of cognitive training and music practice.

Over the past decade, research has shown that WM capacity is subject to training-induced improvements (Klingberg et al., 2002, 2005; Holmes et al., 2009; Thorell et al., 2009; Brehmer et al., 2012; Green et al., 2012) and that the size of the transfer effects are linearly related to practice time (Jaeggi et al., 2008; Bergman Nutley et al., 2011). It is therefore likely that the time spent on other WM taxing activities may affect WM capacity and other abilities partly depending on the same brain regions. Reasoning has also previously reported to improve by training on WM tasks (Klingberg et al., 2002, 2005; Jaeggi et al., 2008), non-verbal reasoning tasks (Bergman Nutley et al., 2011; Mackey et al., 2011), as well as in strategy training studies (Klauer et al., 2002; Hamers et al., 1998). These types of interventions target different aspects of reasoning and the performance improvements reported probably have different origins. Interestingly, it has been shown that it is possible to improve test performance in a single practice session by learning to, e.g., attend to one stimulus dimension at a time (Denney and Heidrich, 1990). This indicates an immediate effect, based on learned strategies, on how to best approach the visuo-spatial structure of the task and is independent of practice time.

The decoding of musical notation is one factor that may explain the effect of music on cognitive functioning as it requires abilities such as spatial–temporal reasoning and visual perception (Gromko, 2004). Given that the results indicate linear effects of music practice on WM and that previous studies have demonstrated effects of music practice on reasoning after as little as 1 year of practice (Rauscher et al., 1997; Schellenberg, 2004), it may be that the music-related processes taxing reasoning are trained while *learning* the musical notation code, whereas the processes taxing WM are trained while *reading* the code, keeping sections of code in WM and in control of attention. This would thus explain the linear relation with time practiced between WM and music, as two more years of music reading would further support development of WM. The effect of music training on reasoning development, on the other hand, may occur as quickly as the foundation of the musical systems are understood rather than linearly and gradual. That being said, regardless of beneficial strategies or basic understanding of the task, WM is required to solve reasoning problems (Carpenter et al., 1990) and to read musical notation (which could explain the trend to linear effects on reasoning development).

Since the music players in this study showed superior performance on cognitive measures already at time point one it is not possible to conclude that music practice caused the cognitive effects, although the dose-response relation indicates that this could be the case. Given that the groups differed in age and that the natural development of the cognitive functions assessed in this study are not linear throughout the age span of the sample, it was not possible to simply test an interaction between time and music in the mixed model. In order to address this, case-control matched analyses were attempted but resulted in too few control cases to enable the analyses at sufficient statistical power. Hence, we instead performed linear regressions with the outcome at later time points as the dependent variable while controlling for the outcome at earlier time points to inform about the change in cognitive ability related to music practice between time points.

Although prior research has suggested positive cognitive effects of physical activity (Hillman et al., 2008; Hotting and Roder, 2013) as well as from playing computer games (Green and Bavelier, 2012), we only found a positive effect for verbal WM development related to time spent on TV viewing/online and gaming activities. The reasons for a lack of a general effect on all cognitive tests are unclear, however, could be due to the crude measures of the covariates (combined score for the gaming/online/TV viewing variable, with no distinction made between verbal and more visuo-spatial activities and no quantification of aerobic fitness or activity type). It may be that the combined score for gaming/online and TV viewing represented activities relying on the use of verbal information processing to a larger extent than taxing the visuo-spatial domain.

There may be alternative explanations to the different trajectories of WM development that in this study appear to be music-related, such as differences in personality. A recent study showed that personality type predicted the duration of music training (along with parental education) to a larger degree than IQ (Corrigall et al., 2013). This could be of importance as our results suggest that time spent on music practice is predictive of WM development and could perhaps partly be explained by the fact that certain personality types of high-functioning individuals tend to show longer durations of practice and may, independently of music, have a different trajectory of their WM development. Another possibility is that there could be an interaction between personality type and the cognitive training effects associated with music practice. This will be for future studies to investigate.

It is likely that part of the sample in our study is musically trained in the vocal domain, which is not accounted for. However, this should hardly affect the interpretation of the results as it is likely to add noise to the data, underestimating the strength of the effects, if anything. Future studies should continue to pursue the question of causality, the mechanism through which music improves cognitive ability as well as potential effects on academic outcome. Ideally, detailed information on musical notation reading skills should be recorded along with personality dimensions and compared with performance on cognitive tests to further investigate the mechanisms involved in music practice and its effects on cognitive ability.

## CONCLUSION

The results from this study show that music training is associated with cognitive and mathematical benefits. This was made apparent through the superior level of performance across time for music players compared with non-players. There was also a difference in the gray matter density within brain in areas related to music notation decoding. Furthermore, the data suggest that music players show a steeper development of both visuo-spatial and verbal WM over time, supporting previously reported causal effects between music practice and cognitive performance. Time spent on music practice predicted both visuo-spatial WM and verbal WM development. More generally, these findings support the importance of practice and learning for the development of WM during childhood and adolescence.

## Conflict of Interest Statement

Sissela Bergman Nutley is an employee of Pearson/Cogmed. Torkel Klingberg has had consultancy agreement with Pearson/Cogmed.
